# Quantitative PA tomography of high resolution 3-D images: Experimental validation in a tissue phantom

**DOI:** 10.1016/j.pacs.2019.100157

**Published:** 2020-01-08

**Authors:** Jens Buchmann, Bernhard Kaplan, Samuel Powell, Steffen Prohaska, Jan Laufer

**Affiliations:** a Institut für Physik, Martin-Luther-Universität Halle-Wittenberg, von-Danckelmann-Platz 3, 06120 Halle (Saale), Germany; b Institut für Optik und Atomare Physik, Technische Universität Berlin, Straße des 17, Juni 135, 10623 Berlin, Germany; c Visual Data Analysis, Zuse Institute Berlin, Takustr. 7, 14195 Berlin, Germany; d Optics and Photonics Group, Faculty of Engineering, University of Nottingham, University Park, Nottingham NG7 2RD, United Kingdom

**Keywords:** Quantitative photoacoustic imaging, Blood oxygen saturation, Inverse problem, Model based inversion, Monte Carlo, Spectral unmixing

## Abstract

Quantitative photoacoustic tomography aims to recover the spatial distribution of absolute chromophore concentrations and their ratios from deep tissue, high-resolution images. In this study, a model-based inversion scheme based on a Monte-Carlo light transport model is experimentally validated on 3-D multispectral images of a tissue phantom acquired using an all-optical scanner with a planar detection geometry. A calibrated absorber allowed scaling of the measured data during the inversion, while an acoustic correction method was employed to compensate the effects of limited view detection. Chromophore- and fluence-dependent step sizes and Adam optimization were implemented to achieve rapid convergence. High resolution 3-D maps of absolute concentrations and their ratios were recovered with high accuracy. Potential applications of this method include quantitative functional and molecular photoacoustic tomography of deep tissue in preclinical and clinical studies.

## 1 Introduction

Photoacoustic (PA) tomography is an emerging imaging modality that combines the high contrast and spectral specificity of purely optical imaging methods with the high spatial resolution of ultrasound [1], [2]. It relies on the absorption of short optical pulses by tissue chromophores to generate broadband acoustic fields. The spatial distribution of the optical absorbers within the illuminated volume is therefore encoded onto the time-resolved pressure signals measured at multiple locations outside the imaged object. From this data, 3-D images of the initial pressure distribution, which depends on the local abundance of all tissue chromophores, are then reconstructed. Quantitative photoacoustic tomography (QPAT) aims to recover the spatial distribution of absolute chromophore concentrations, such as oxy- and deoxyhemoglobin as well as contrast agents, from these images to extend the capabilities of this modality to quantitative, deep-tissue functional and molecular imaging. To reach this goal, a number of key challenges have to be addressed. The accurate prediction of the spatially varying and wavelength-dependent fluence, which causes so-called spectral coloring and structural corruption [3], is vital. Thermodynamic properties, such as the dependence of the Grüneisen parameter on chromophore concentration, also need to be accounted for. In addition, factors such as limited view detection, scanner-specific instrument transfer functions and detection noise determine to which extent a measured PA image represents the true initial pressure distribution. Lastly, experimental and computational methods are required to address the large scale of the inverse problem posed by high resolution images, which can result in millions of variables [3], [4], [5], [6].

A variety of QPAT methods have been used to invert measured PA images. Early fluence correction methods, which used linear inversions under the assumption of homogeneous optical properties and empirical parameters to obtain images of relative concentrations [7], [8], have been shown to be severely limited [9], [10]. Other studies based on linear inversions incorporated independent measurements using acousto-optics [11], [12] or diffuse optical tomography [13], [14], [15] to obtain an estimate of the fluence. Data-driven methods, by contrast, estimate the fluence using algorithms trained on a large number of data [16], [17]. However, obtaining sufficiently large training data through measurements is often impractical, and the use of models to simulate images acquired using real imaging systems is non-trivial. It is therefore not yet clear to which extent these methods are generally applicable.

Model-based inversion schemes are one of the most promising and potentially generally applicable methods for QPAT due to the flexibility numerical models afford. In principle, all physical processes involved in the generation of a PA image can be represented. By expressing a forward model as a function of specific input parameters, e.g. chromophore concentrations and optical scattering, its output can be fitted to measured data using, for example, a least-squares minimization technique. The set of parameter values determined this way is then considered that which has the highest likelihood of explaining the experimental results. Most studies in this field have relied on the diffusion approximation as a light transport model. It has the advantage that it is easily implemented but lacks accuracy in non-diffuse illumination, e.g. close to the optical source. It was nevertheless shown to enable the recovery of absolute concentrations and a global scattering coefficient from multispectral 2-D images given prior knowledge of the wavelength dependence of the molar absorption and of scattering [9], [18]. Fixed-point iteration schemes have been applied in 2-D and 3-D [19], [20] but require *a priori* knowledge of the scattering distribution and quickly diverge if the scattering coefficient is wrong [3]. Recently, an iterative optimization scheme for the fluence correction of 2-D images taken with a commercially available scanner was reported [21], [22]. While 2-D images are generally insufficient to support QPAT of complex 3-D objects, the method also relies on manual image segmentation to enable stable inversions.

The efficient calculation of functional gradients is a vital capability, particularly if the scale of the inverse problem reaches millions of unknowns [23], and has been demonstrated using a diffusion light transport model [24], [25]. An iterative method for inverting high-resolution images *in silico* using a Monte-Carlo light transport model was recently reported by the authors [26]. While this method is – in principle – generally applicable, the inversion of measured images poses additional challenges since image reconstruction artifacts, limited detection apertures, and noise cause an inherent mismatch between the model output and the experimental data.

In this work, methods for minimizing this mismatch were developed and applied to images acquired in a tissue-mimicking phantom to recover absolute chromophore concentrations and concentration ratios from high resolution 3-D PA images. A Monte-Carlo (MC) light transport model was used to obtain accurate predictions of the fluence for superficial imaging depths (up to 1 cm). Multispectral image data sets were obtained in planar detection geometry using a Fabry–Pérot based PA scanner [27], [28]. To account for the partial mapping of the PA field due to the limited detection aperture and its effect on the reconstructed images, an *ad hoc* correction method was employed. To accurately scale the image intensity of the measured data sets, a calibrated absorber was used, and the concentration dependence of the Grüneisen parameter was incorporated into the forward model. The Adam algorithm [29] enabled an efficient inversion despite noisy gradients. While this methodology allows the simultaneous inversion of absorption and scattering coefficients in principle, the scattering distribution was assumed to be known *a priori* in this study.

In Section 2, the methods are introduced. This includes the forward model (Section 2.1), the experimental setup and tissue phantom (Section 2.2), the limited view correction method, and further details of the optimization (Section 2.3). In Section 3 the inversion results are presented and limitations of the method are discussed. Concluding remarks are given in Section 4.

## 2 Methods

### 2.1 Model-based inversion scheme

#### 2.1.1 PA forward model

The intensity of a PA image is considered proportional to the initial pressure distribution, *p*_0_, which is given by

(1) $$p_{0}(r,\lambda )=\Gamma (r)H(r)=\Gamma (r)\mu_{a}(r,\lambda )\Phi (r,\lambda ),$$

where Γ is the Grüneisen parameter, which describes the PA efficiency, *H* is the absorbed energy density, *μ*_*a*_ is the absorption coefficient, Φ is the light fluence, r is the position, and *λ* is the excitation wavelength. $p_{0}(r,\lambda )$ is referred to as (PA) image data set throughout this paper. The wavelength-dependent absorption coefficient at position r due to the presence of *k* different chromophores is a function of the specific absorption coefficients, *α*_*k*_(*λ*), and concentrations $c_{k}(r)$ as given by

(2) $$\mu_{a}(\lambda ,r)=\sum_{k}^{N_{k}}c_{k}(r)\alpha_{k}(\lambda ),$$

where *N*_*k*_ is the number of chromophores. The Grüneisen parameter and its concentration dependence was approximated using

(3) $$\Gamma (r)=\Gamma_{\mathrm{water}}\left(1+\sum_{k}\beta_{k}c_{k}(r)\right),$$

where Γ_water_ is the Grüneisen parameter of water and *β*_*k*_ are empirical, chromophore-dependent coefficients [18], [30], [31], [32]. To calculate a PA image data set, i.e. $p_{0}(r,\lambda )$, a Monte Carlo (MC) model was used to predict the fluence, Φ(r,λ) [33], [34]. The input parameters were the illumination geometry, i.e. the incident laser beam profile and its location, the chromophore concentrations, the scattering coefficients, and the refractive index distribution.

#### 2.1.2 Iterative inversion scheme

The iterative inversion scheme is described in detail elsewhere [26]. Briefly, the spatial distribution of the incident excitation pulses, the refractive index, and the scattering coefficient distribution were assumed to be known *a priori* and remained constant during the inversion. The MC model was initialized assuming homogeneous absorption by water and run to calculate a PA image data set, $p_{0}(r,\lambda )$. The difference between $p_{0}(r,\lambda )$ and the measured PA image data set, $p_{0}^{m}(r,\lambda )$, which was reconstructed from the measured sensor-time series, $p^{m}(x,t,\lambda )$, was represented by a least-squares error functional, *ε*, as given by

(4) $$\varepsilon =\sum_{l}^{N_{\lambda }}\int_{\Omega }\frac{1}{2}{\left[{\mathrm{Kp}}_{0}^{m}(r,\lambda_{l})-p_{0}(r,\lambda_{l})\right]}^{2}d\Omega ,$$

where *K* is a scaling factor (described in Section 2.3.2), *N*_*λ*_ is the number of excitation wavelengths *λ*_*l*_ and Ω is the image volume. By minimizing *ε*, 3-D maps of chromophore concentrations were recovered from measured image data sets. The gradient of *ε* with respect to the concentration of chromophore *c*_*k*_ at position $r_{i}$ is given as

(5) $$\frac{\partial \varepsilon }{\partial c_{k}(r_{i})}=-\frac{1}{N_{\lambda }}\sum_{l}^{N_{\lambda }}\left[{\mathrm{Kp}}_{0}^{m}(r_{i},\lambda_{l})-p_{0}(r_{i},\lambda_{l})\right]V_{\mathrm{vox}}\left[\beta_{k}H(r_{i},\lambda )+\Gamma (r_{i})\alpha_{k,l}\Phi (r_{i},\lambda_{l})\right],$$

where *V*_vox_ is the volume of a single image voxel. It should be noted that this gradient is an approximation. It was found to be valid for inversions where the scattering coefficient can be assumed known and fixed [26]. The chromophore concentrations, the gradients, and the chromophore- and fluence-dependent step size were updated at each iteration (described in Section 2.3.4).

### 2.2 Data acquisition

#### 2.2.1 Experimental setup

**Fig. 1:**
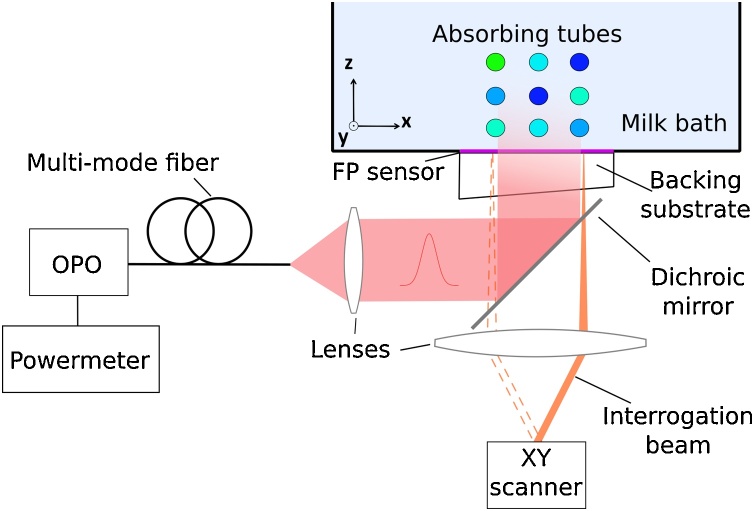
Schematic of the experimental setup. Excitation pulses were generated by a wavelength-tunable OPO laser, the output of which was fiber-coupled to homogenize the beam. The output of the distal end of the fiber was collimated and directed through the FP sensor using a dichroic mirror.

The experimental setup is shown in Fig. 1. A custom-built Fabry–Pérot (FP) based PA scanner with planar detection geometry was used to acquire multispectral PA image data sets [27], [35]. The thickness of the Fabry–Perot polymer film sensor was 40 μm, resulting in a flat acoustic frequency response from 50 kHz to 20 MHz. Due to the small element size of 60 μm, the response of the sensor is near omni-directional [36]. An OPO laser (Spitlight 1000, Innolas GmbH) with a pulse repetition frequency of 30 Hz was used as an excitation source. Its output was coupled into a 1.5 mm dia. multimode fiber to homogenize the beam. The collimated fiber output illuminated the phantom through the FP sensor in a backward-mode imaging configuration. The beam profile, which exhibited a near Gaussian profile (1/*e*^2^ dia. ≈ 13 mm), was determined from the maximum intensity projection of a PA image of a water-coupled neutral density filter that was in close proximity to the sensor.

PA image data sets were acquired by 2-D raster-scanning the focused beam of a cw interrogation laser (Tunics T100s, Yenista) across the FP sensor (16 mm × 16 mm with 110 μm increments) and by recording the time-resolved modulation of the reflected optical power, i.e. the PA signal, at each scan position. Images were acquired at five excitation wavelengths (i.e. 688, 721, 765, 811, 867 nm) and were reconstructed using the time-reversal method of the k-Wave toolbox [37]. The speed of sound was determined from the measured images using an auto-focus function [38] and was found to be 1499 m/s. The intensity of the images was normalized to correct for the wavelength dependent pulse energy with an incident fluence ranging from 5 to 15 mJ/cm^2^ at the surface of the phantom.

#### 2.2.2 Tissue phantom

**Fig. 2:**
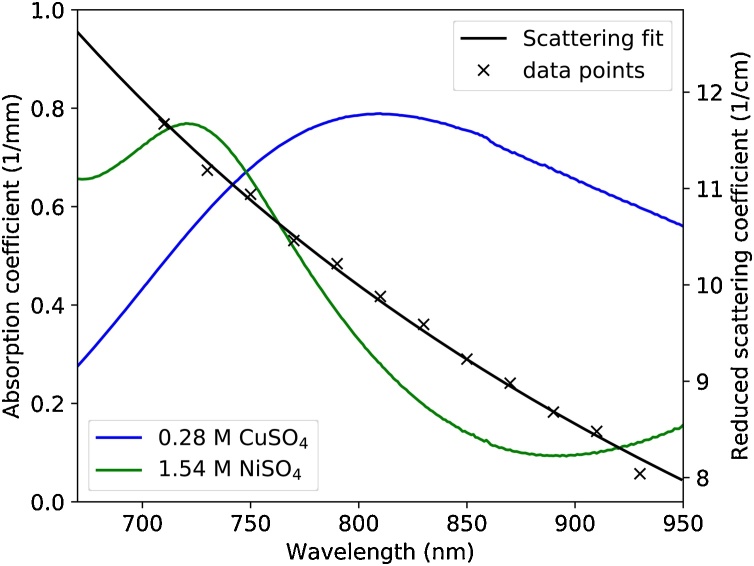
Absorption spectra of aqueous stock solutions of NiSO_4_ (green) and CuSO_4_ (blue) with concentrations of 1.54 and 0.28 M, respectively. Black crosses indicate the measured reduced scattering coefficient of diluted whole milk, the black line shows the exponential fit to the data.

**Fig. 3:**
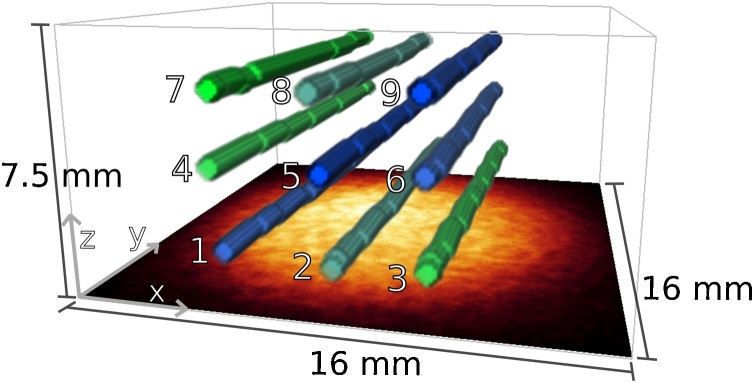
3-D rendered structure of the phantom obtained using manual image segmentation of the PA images. The color of the tubes indicates the relative concentrations, i.e. green indicates $c_{{\mathrm{NiSO}}_{4}}$, blue indicates $c_{{\mathrm{CuSO}}_{4}}$. The illumination beam profile of the excitation pulses is shown in the *x*–*y*-plane.

The tissue-mimicking phantom consisted of nine polymer tubes (THV, Paradigm Optics, Inc.) with an inner diameter of 670 μm and an outer diameter of 800 μm. The tubes were immersed in diluted whole milk (3.5% fat) with a milk-to-water ratio of 1:3. The scattering coefficient was measured using time-resolved transmittance spectroscopy [39] at wavelengths between 710 and 890 nm. To interpolate the data, an empirical function for the wavelength dependence of scattering ($\mu_{s^{\prime }}(\lambda )=a\cdot \lambda_{+}^{b}\;{\mathrm{mm}}^{-1}$ with *λ*_+_ = *λ*/nm) was fitted to the measured spectrum. The best fit yielded *a* = 6.65 × 10^3^ and *b* =−1.317. The anisotropy factor *g* was assumed to be 0.9. To mimic the optical absorption of oxy- and deoxyhemoglobin, mixtures of aqueous stock solutions of copper and nickel sulfate (CuSO_4_ and NiSO_4_) were prepared to fill the tubes. The concentrations of the CuSO_4_ and NiSO_4_ stock solutions were 0.28 and 1.54 M, respectively. CuSO_4_ and NiSO_4_ are ideal chromophores for PA tissue phantoms because they are photostable during high peak power illumination and their absorption spectra mix linearly [31]. The concentration- and chromophore-dependence of the Grüneisen parameter was described using Eq. (3) and empirically determined coefficients ($\beta_{{\mathrm{CuSO}}_{4}}=0.708\;M^{-1}$, $\beta_{{\mathrm{NiSO}}_{4}}=0.325\;M^{-1}$) [31]. Γ_water_ was set to 0.124 [40], [31]. The tubes were mounted parallel to each other at depths of approximately 2, 4 and 7 mm and were filled with mixtures of the CuSO_4_ and NiSO_4_ solutions at different concentration ratios. The concentration ratio *R* represented a blood oxygen saturation (SO_2_) analogue and was given by

(6) $$R=\frac{c_{{\mathrm{NiSO}}_{4}}/1.54}{c_{{\mathrm{NiSO}}_{4}}/1.54+c_{{\mathrm{CuSO}}_{4}}/0.28},$$

i.e. the concentrations of mixed solutions were normalized to the molar concentration of the stock solutions. Spectra of the absorption coefficient of the solutions and the reduced scattering coefficient are shown in Fig. 2. Fig. 3 shows a 3-D rendering of the phantom structure, which was extracted from the measured PA images using a mixture of manual image segmentation and a slice-wise template-matching algorithm to identify the center position of each tube.

### 2.3 Image correction and inversion details

#### 2.3.1 Limited view correction

For a truthful acoustic reconstruction of *p*_0_, a detection surface angle of greater than 2*π* steradian is required [41], [42]. Since a finite detection aperture, such as the planar aperture of the all-optical FP scanner, limits the surface angle, the reconstructed images exhibit the effects of partial data, such as erroneous image intensities and artifacts. This can reduce the accuracy of concentrations recovered using inversion schemes. Limited aperture effects can nevertheless be compensated to some extent by either correcting the measured images using approximate methods or by incorporating a numerical model of acoustic propagation and detection into the PA forward model. In this study, an *ad hoc* method based on the calculation of a correction matrix, which represents the ratio of images acquired using limited and full aperture detection, was applied to account for the loss of information. The calculation of the correction matrix required the following steps: First, the measured PA image data set, $p_{0}^{m}(r,\lambda )$, was smoothed using the built-in smooth function (k-Wave) [37] to reduce effects of detection noise. To avoid division by zero during subsequent steps, intensities below a threshold of 1/100 of the maximum *p*_0_ were set to the threshold value. The smoothed and thresholded data set is referred to as $p_{0,\mathrm{smooth}}^{m}(r,\lambda )$. Second, $p_{0,\mathrm{smooth}}^{m}(r,\lambda )$ was used as an initial pressure distribution in a 3-D acoustic propagation model (k-Wave) to obtain a PA time series $p_{\mathrm{fw}}^{m}(x,t,\lambda )$ detected over an aperture identical to that of the planar FP sensor with which $p_{0}^{m}(r,\lambda )$ was originally acquired. x indicates the detector positions. From $p_{\mathrm{fw}}^{m}(x,t,\lambda )$, the PA image data set $p_{0,\mathrm{fw}/\mathrm{bw}}^{m}(r,\lambda )$ was reconstructed. $p_{0,\mathrm{fw}/\mathrm{bw}}^{m}(r,\lambda )$ exhibits different (lower) image intensities compared to the original $p_{0}^{m}(r,\lambda )$. This difference can be used to approximate the error caused by the limited detection aperture. Third, the correction matrix, η(r), was obtained by dividing the two data sets and averaging over all wavelengths:

(7) $$\eta (r)=\frac{1}{N_{\lambda }}\sum_{\lambda }\frac{p_{0,\mathrm{fw}/\mathrm{bw}}^{m}(r,\lambda )}{p_{0,\mathrm{smooth}}^{m}(r,\lambda )}.$$

Finally, the measured PA images were corrected using

(8) $$p_{0,\mathrm{corrected}}^{m}(r,\lambda )=p_{0}^{m}(r,\lambda )/\eta (r).$$

By averaging *η* over all wavelengths, it was ensured that the spectral information of the image data set was not distorted.

#### 2.3.2 Calibrated absorber

Measured PA image intensities, i.e. $p_{0}^{m}(r_{i},\lambda )$, are rarely presented in units of absolute pressure as the calibration of an imaging system is challenging. It is nevertheless advantageous to scale measured images to the output of a model to achieve an efficient and accurate inversion. In this study, a calibrated absorber positioned within the image volume (tube 2 in Fig. 3) was used as a reference with which the measured images were scaled to those calculated using the forward model. The optical and acoustic properties of the calibrated absorber, such as *μ*_*a*_(*λ*) and Γ, were known *a priori*. The sub-volume of the calibrated absorber was segmented manually, and the corresponding *μ*_*a*_(*λ*) and Γ remained fixed during the inversion. The scaling factor *K* was updated after every iteration using

(9) $$K=\sum_{\lambda }\sum_{r_{\mathrm{CA}}}\frac{\Gamma H_{0}(r_{i},\lambda )}{p_{0}^{m}(r_{i},\lambda )},$$

where $r_{\mathrm{CA}}$ indicates the sub-volume of the calibrated absorber. Using *K*, the measured data were scaled according to Eq. (4).

#### 2.3.3 Monte Carlo light model

5 × 10^6^ photon packets were launched to simulate a PA image $p_{0}(r,\lambda )$ at a single excitation wavelength. The domain of the model was discretized into isotropic voxels with a width of 110 μm. The model domain was extended to 300 × 300 × 150 voxels compared to the size of the measured data (146 × 145 × 75 voxels) to ensure the correct calculation of optical scattering beyond the boundaries of the measured image [43]. In the extended volume, water was assumed to be the sole chromophore. The internal refractive index was set to that of water (1.33). The external refractive index was set to 1.5 to match that of the FP sensor.

#### 2.3.4 Optimization

The gradients of the error functional, *ε*, (Eq. (4)), are affected by noise due to the stochastic nature of the MC model. This makes standard gradient descent methods unsuitable because the noise increases the likelihood of convergence to a local minimum or saddle point. To overcome this limitation, the Adam algorithm was employed. It is an extension of the momentum gradient descent specifically designed to deal with noisy gradients [29]. In this study, most parameters of the algorithm, except for the step size *γ*, were set to the value suggested by the creators of the algorithm. *γ* was preconditioned for stable and fast convergence [26] by making it dependent upon the type of chromophore and the fluence. To account for the differences in the specific absorption coefficients of the three chromophores (NiSO_4_, CuSO_4_, H_2_O), *γ*_k_ for CuSO_4_ was set *ad hoc* to *γ*_Cu_ = 0.1, and the step size for the remaining two chromophores was calculated using

(10) $$\gamma_{k}=\gamma_{\mathrm{Cu}}\frac{\sum_{\lambda }\alpha_{k,\lambda }\cdot c_{\max,\;\mathrm{Cu}}}{\sum_{\lambda }\alpha_{\mathrm{Cu},\lambda }\cdot c_{\max,\;k}},$$

where *k* ∈ {CuSO_4_, NiSO_4_, H_2_O}, and *α*_*k*,*λ*_ represents the specific absorption coefficients of NiSO_4_ and CuSO_4_ and the absorption coefficient of water at wavelength *λ*. *c*_max,k_ are maximum concentrations of the chromophores and were set to *c*_max,Cu_ = 0.28 M, *c*_max,Ni_ = 1.54 M, and $c_{{\max,H}_{2}O}=1$. It is worth mentioning that in cases where the maximum concentrations of the chromophores are not known, rough estimates are also sufficient. Lastly, the step size at each location r was expressed as a function of the local fluence using

(11) $$\gamma_{k,\mathrm{scaled}}(r)=\frac{\gamma_{k}}{{\tilde{\Phi }}_{\mathrm{norm}}(r)+\varepsilon_{\Phi }},$$

where ${\tilde{\Phi }}_{\mathrm{norm}}(r)$ is the normalized fluence, averaged over all wavelengths. *ε*_Φ_ is a fixed-value parameter to avoid division by zero (*ε*_Φ_ = 10^−4^). The range of chromophore concentrations was restricted to plausible values, i.e. only non-negative concentrations were allowed and upper bound constraints of NiSO_4_ and CuSO_4_ were set to their solubility (2.36 and 1.28 M, respectively). The water content was allowed to vary between 0 and 1. The inversion scheme was run for 300 iterations on both the original PA image data sets and those corrected using the methods described in Section 2.3.1.

## 3 Results and discussion

### 3.1 Comparison of original and corrected PA images

**Fig. 4:**
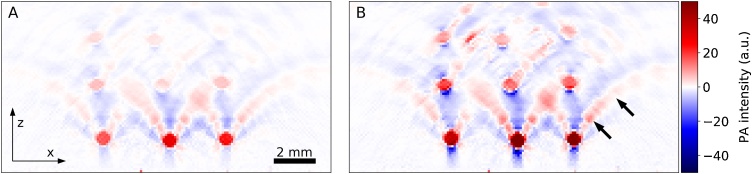
Cross-sectional *x*–*z*-images (*y* = 8 mm) of the tissue phantom at an excitation wavelength of 721 nm. (A) Original image and (B) after applying the limited view correction (Section 2.3.1).

Cross-sectional *x*–*z*-images of the measured 3-D PA images before and after applying the limited view correction method are shown in Fig. 4. The FP sensor plane is at the bottom of the images in the *xy*-plane. A center *xz*-slice at *y* = 8 mm of an original PA image is shown in Fig. 4A (*λ*_*exc*_ = 726 nm, normalized to 1 J incident pulse energy). Fig. 4B shows the result of the limited view correction (Eq. (7)). The arc-shaped features indicated by black arrows in Fig. 4B are artifacts that result from the limited planar detection geometry. While the limited view correction increased the image intensity in general, the intensity of the deeper lying objects was amplified more strongly than those located at shallow depths. The signals of the shallow tubes were doubled in amplitude by the limited view correction, while the amplitudes of the deepest row of tubes were increased by a factor of 3–5.

### 3.2 Convergence

The inversion was found to fully converge within 300 iterations after 8 h computation time using an NVIDIA Titan X (Pascal) GPU. The use of chromophore- and fluence-dependent step sizes (Eqs. (10) and (11)) produced an increase in convergence speed of more than an order of magnitude due to a better efficiency in regions of low fluence. Importantly, inversions without the step size correction were found to fail.

### 3.3 Recovered chromophore concentrations

**Fig. 5:**
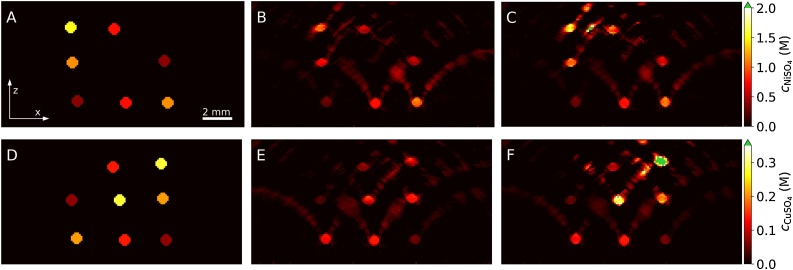
Cross-sectional *x*–*z*-images (*y* = 8 mm) of the true and recovered chromophore concentrations. (A) and (D) True $c_{{\mathrm{NiSO}}_{4}}$ and $c_{{\mathrm{CuSO}}_{4}}$, respectively. (B) and (E) $c_{{\mathrm{NiSO}}_{4}}$ and $c_{{\mathrm{CuSO}}_{4}}$ recovered from uncorrected images. (C) and (F) $c_{{\mathrm{NiSO}}_{4}}$ and $c_{{\mathrm{CuSO}}_{4}}$ recovered from corrected images.

The recovered chromophore concentrations are shown in Fig. 5. Fig. 5A and D illustrates the true distribution of $c_{{\mathrm{NiSO}}_{4}}$ and $c_{{\mathrm{CuSO}}_{4}}$, Fig. 5B and E shows the concentrations recovered from the original image data set, and Fig. 5C and F show those from the aperture-corrected data set. Several observations can be made. First, significant $c_{{\mathrm{NiSO}}_{4}}$ and $c_{{\mathrm{CuSO}}_{4}}$ were recovered in the background sub-volume even though this region contained only water. This is explained by image reconstruction artifacts, which lead to significant recovered concentrations in regions coinciding with positive-going artifacts. Second, the circular tube cross-sections are distorted in the deeper lying tubes, which is due to the limited detection aperture. The concentrations recovered for the most superficial tubes (including the calibrated absorber, bottom center) are largely unaffected by the acoustic correction. This is in strong contrast to the deeper lying tubes where significantly higher concentrations were recovered from the corrected image data set compared to those obtained from the original. This also resulted in a better agreement with the true values (Fig. 5A and D). However, the increase in the absolute values also applied to concentrations recovered from the background, i.e. regions corresponding to image artifacts.

**Fig. 6:**
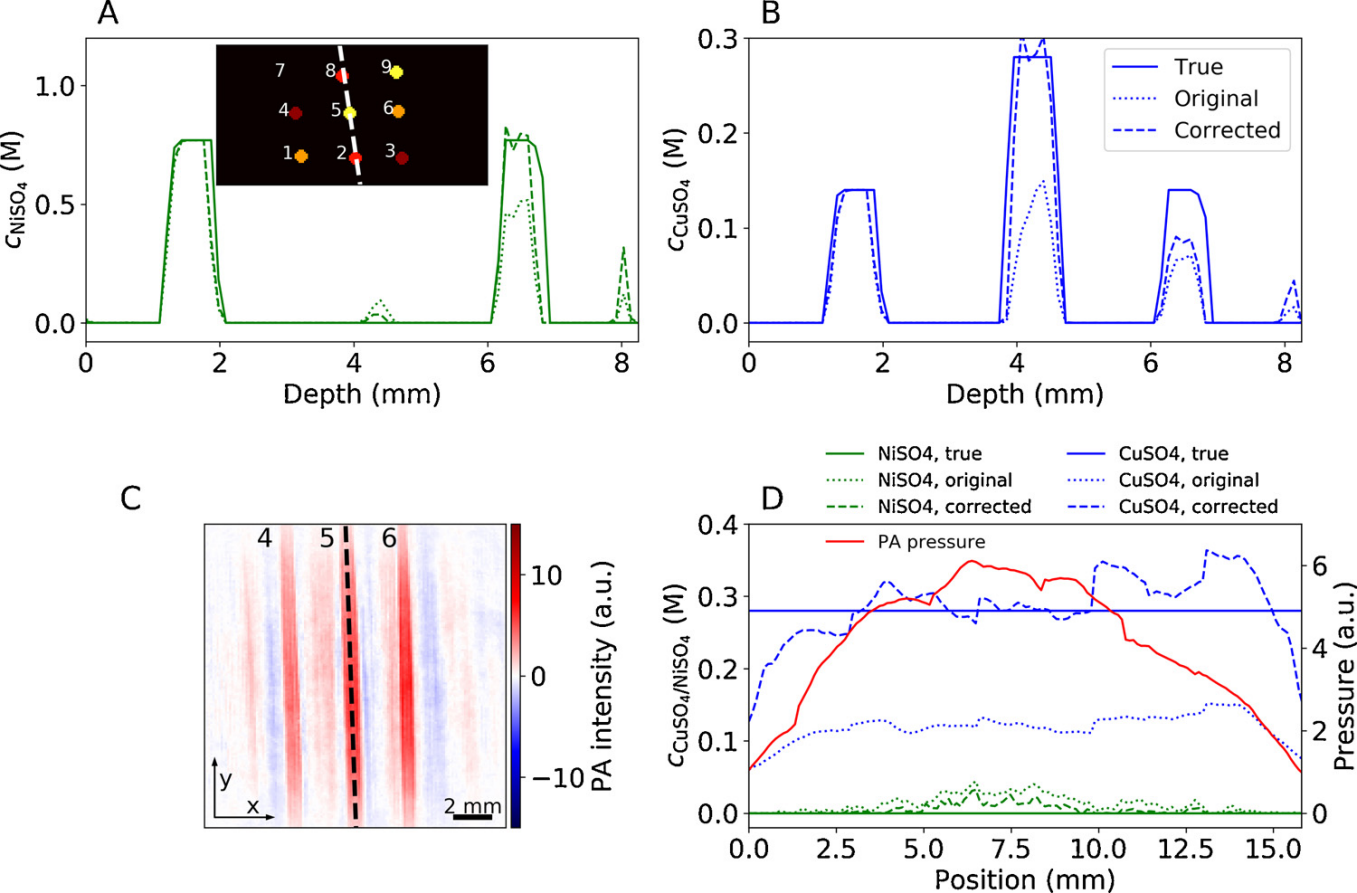
Depth profile of the true and recovered 3-D maps of $c_{{\mathrm{NiSO}}_{4}}$ (A) and $c_{{\mathrm{CuSO}}_{4}}$ (B) along the dashed line indicated in the inset of (A). Inset of (A) *x*–*z*-slice of the true $c_{{\mathrm{CuSO}}_{4}}$ distribution (see Fig. 5D). (C) cross-sectional *x*–*y*-image of the initial pressure distribution at depth of *z* = 4.2 mm (*λ* = 726 nm). The initial pressure along the dashed line is shown in (D), which also shows the chromophore concentrations recovered from original and corrected image data sets. The solid, dotted and dashed lines indicate the true values, those recovered from original images, and those recovered from corrected images, respectively. $c_{{\mathrm{NiSO}}_{4}}$ is shown in green and $c_{{\mathrm{CuSO}}_{4}}$ in blue. The red solid line illustrates the initial pressure distribution (original image data set).

Line profiles through the recovered concentration maps are shown in Fig. 6. The profiles were extracted from the 3-D maps along the dashed line in the inset of Fig. 6A, which intersects the locations of three tubes (tube IDs 2, 5 and 8). The profiles of $c_{{\mathrm{NiSO}}_{4}}$ and $c_{{\mathrm{CuSO}}_{4}}$ are shown in Fig. 6A and B. The solid lines depict the true concentrations, the dotted lines the concentrations recovered from the original images and the dashed lines those recovered from limited view corrected images. The first peak at a depth of approximately 2 mm corresponds to the tube that served as a calibrated absorber, showing perfect agreement between the true and recovered values. A small second peak in $c_{{\mathrm{NiSO}}_{4}}$ (Fig. 6A) can be found at around 4 mm depth, which corresponds to the location of tube 5 in which $c_{{\mathrm{NiSO}}_{4}}=0$ M. By contrast, the high $c_{{\mathrm{CuSO}}_{4}}$ within tube 5 is clearly visible in Fig. 6 B. The peaks at a depth of 6–7 mm in Fig. 6A and B correspond to the location of tube 8, which contained a mixture of CuSO_4_ and NiSO_4_. In general, the concentrations recovered from the original image data set are significantly lower than the true values. By contrast, good agreement was observed for the corrected images. The peak in $c_{{\mathrm{NiSO}}_{4}}$ and $c_{{\mathrm{CuSO}}_{4}}$ at a depth of approximately 8 mm corresponds to an image reconstruction artifact.

Fig. 6C shows a cross-sectional *xy*-image of an original $p_{0}^{m}(r,\lambda )$ at a depth of *z* = 4.2 mm (*λ*_exc_ = 726 nm). While the tubes 4, 5 and 6 are clearly shown, image reconstruction artifacts are also noticeable. The profiles of the $c_{{\mathrm{NiSO}}_{4}}$ and $c_{{\mathrm{CuSO}}_{4}}$ along the length of tube 5 (corresponding to the dashed line in Fig. 6C) are shown in Fig. 6D together with that of the initial pressure distribution to illustrate the effectiveness of the fluence correction provided by this method. The dashed and dotted green and blue lines represent $c_{{\mathrm{NiSO}}_{4}}$ and $c_{{\mathrm{CuSO}}_{4}}$, respectively, recovered from the original and corrected image data sets (averaged over the tube cross-section). The solid red line illustrates the initial pressure distribution (original image data set), which is six times greater at the center of the image than at the boundary. By contrast, the values of the recovered concentrations show much smaller variations along the length of the tube, demonstrating that the inversion scheme is highly effective at compensating the fluence variation. A deviation of the recovered from the true values was observed at the edge of the imaged volume (1–2 mm from the image boundary). This may be explained by the inherently low SNR in this region and the potential limitations in estimating the backscattering of light from adjacent regions into the image volume using the extended MC model. Importantly, the chromophore concentrations determined from the corrected image data set are much closer to the true values compared to those obtained from the original images.

The concentrations recovered from each sub-volume of the phantom are listed in Table 1. It was found that the concentration of water had a negligible effect on the PA pressure, which is explained by its low absorption coefficient at the excitation wavelengths used in this study. This resulted in low sensitivity and accuracy for the recovered water concentrations. On a positive note, the errors in the water concentration were found to have a negligible effect on the accuracy of the recovered $c_{{\mathrm{NiSO}}_{4}}$ and $c_{{\mathrm{CuSO}}_{4}}$.

**Table 1:** Absolute chromophore concentrations and their ratio *R* recovered using the model-based inversion (BKG = background, Orig = original images, Corr = acoustically corrected images). Values in brackets indicate the standard deviation (concise notation) within the sub-volume.

Tube ID	$c_{{\mathrm{NiSO}}_{4}}$ [M]			$c_{{\mathrm{CuSO}}_{4}}$ [M]			*R* [%]		
	True	Orig	Corr	True	Orig	Corr	True	Orig	Corr
BKG	0	0.02(7)	0.02(9)	0	0.003(11)	0.004(23)	n/a	51.1	44.4
1	0.385	0.25(6)	0.26(7)	0.21	0.13(3)	0.13(3)	25	25.8	25.5
2	0.77	0.77(0)	0.77(0)	0.14	0.14(0)	0.14(0)	50	50.0	50.0
3	1.154	1.14(26)	1.18(29)	0.07	0.05(1)	0.05(1)	75	80.7	80.2
4	1.154	0.60(15)	0.97(24)	0.07	0.03(1)	0.04(1)	75	78.1	80.7
5	0	0.012(27)	0.005(19)	0.28	0.12(4)	0.28(8)	0	1.9	0.3
6	0.385	0.41(13)	0.54(19)	0.21	0.13(3)	0.21(5)	25	36.7	32.4
7	1.539	0.80(28)	1.29(39)	0	0.001(3)	0.001(4)	100	99.0	99.4
8	0.77	0.39(17)	0.76(30)	0.14	0.06(2)	0.10(3)	50	55.2	58.5
9	0	0.002(10)	0.000(0)	0.28	0.07(4)	0.33(2)	0	0.5	0.0

### 3.4 Concentration ratios

**Fig. 7:**
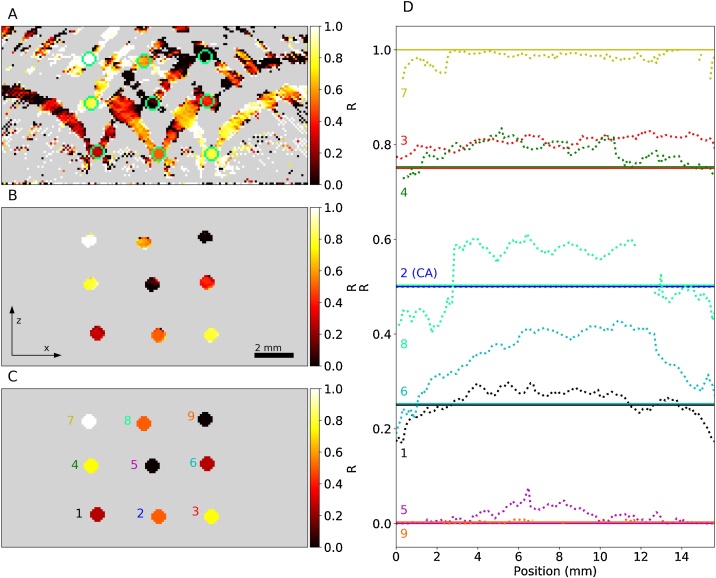
Chromophore ratio *R* calculated from the recovered $c_{{\mathrm{NiSO}}_{4}}$ and $c_{{\mathrm{CuSO}}_{4}}$ maps. (A) Cross-sectional *x*–*z*-image of the recovered concentration ratio *R*, (B) recovered *R*, background subvolume is masked and rendered in gray, (C) true concentration ratios. Gray indicates areas where *R* is not defined, i.e. $c_{{\mathrm{NiSO}}_{4}}=c_{{\mathrm{CuSO}}_{4}}=0$ M. (D) Profiles of *R* (cross-sectional average) along the length of the nine tubes. True values are represented by dashed lines, recovered values by dotted lines.

In Fig. 7, cross-sectional *x*–*z*-images of the true and recovered maps of the concentration ratio *R* are shown. Fig. 7C illustrates the true distribution, which ranges from 0 to 1. In regions where NiSO_4_ and CuSO_4_ were not present, Eq. (6) is not defined (division by zero) and the corresponding regions are rendered in gray. Fig. 7A shows *R* recovered from the original image data set. Due to the image reconstruction artifacts, significant $c_{{\mathrm{NiSO}}_{4}}$ and $c_{{\mathrm{CuSO}}_{4}}$ were recovered across large regions of the background. It was also observed that the value of *R* determined at the location of an artifact is similar to that of the specific absorber, i.e. a tube, where it originated. To aid visualization, the location of the tubes is indicated by green circles in Fig. 7A. Fig. 7B shows the inversion result (Fig. 7A) after masking the background to allow a better comparison of the recovered *R* with the true values (Fig. 7C). In Fig. 7D, profiles of the cross-sectional average of *R* along the length of the nine tubes are shown. For most tubes, *R* is close to the true value and deviates little for the entire length of the tube, including near the image boundaries. The strongest deviation is noticeable for tube 6, where *R* is significantly greater at the center of the image compared to the boundaries. This may be explained by the presence of an arc-shaped artifact that originates from the strong PA signal of tube 2. It is superimposed onto the region that corresponds to tube 6, resulting in a corruption of its PA spectrum. Since tube 2 contained a solution with higher *R* than that of tube 6, the superposition of the PA spectra may explain the error. In general, most values for *R* are slightly higher than their true values (see also Table 1). The reason for this systematic error is unclear. While the limited view correction was found to have a major impact on the recovered absolute chromophore concentrations, its effect on the accuracy of the recovered *R* was negligible. A possible explanation is the absence of significant background absorbers in this specific phantom. In a scenario where the background also contains chromophores at significant concentrations, any change in the image intensity, e.g. from the acoustic *ad hoc* correction, may in turn affect R in deeper lying vessels.

### 3.5 Limitations and general applicability

The method described in this paper was tailored to the inversion of multispectral PA image data sets acquired over a planar detection aperture. It is a flexible approach that allows the inversion of multispectral 3D PA images acquired using constant illumination geometry and is applicable to other targets, scan geometries, and experimental setups. The required *a priori* knowledge does not result in undue restrictions. For example, the chromophores that are expected within the image volume, as well as their absorption spectra and approximate maximum concentrations, are typically known. Similarly, the Grüneisen parameter and its concentration dependence is known for most endogenous tissue chromophores or can be measured. While the use of a calibrated absorber results in additional image processing steps, such as segmentation, it was found to greatly improve the efficiency and accuracy of the inversion. The *ad hoc* acoustic correction was found to compensate the corrupting effects of limited-view detection geometries with sufficient accuracy. The effects of inhomogeneities in the speed of sound and refractive index were not considered in this work but strategies for addressing these effects can be found elsewhere [6]. The assumption of *a priori* knowledge of the wavelength dependence of the scattering coefficient, which is required to solve the non-uniqueness problem of QPAT [44], and its magnitude is a limitation of the current implementation of the method. In theory, the method can be extended to facilitate inversions that recover the spatial distribution of the scattering coefficient (in addition to the chromophore maps) from multispectral images by solely relying on *a priori* knowledge of the scattering wavelength dependence. The inversion of the scattering coefficient may also be achieved by incorporating multiple illuminations in the image acquisition protocol. Lastly, a parameter that currently requires manual optimization when the method is applied to a new set of measurements is the chromophore-dependent step size to minimize the number of iterations needed for convergence.

## 4 Conclusions

In this study, an iterative model-based inversion scheme was successfully used to recover maps of the absolute chromophore concentrations and their ratios from high resolution 3-D PA image data sets measured in a tissue phantom. The forward model, which was based on a MC light transport model, was used to predict 3-D images of the initial pressure distribution. To address the mismatch between the image data sets predicted by the model and those measured using an all-optical PA scanner based on a planar Fabry–Pérot ultrasound sensor, an *ad hoc* correction method was introduced to compensate the effects of limited view detection. A calibrated absorber was used to scale measured data to the output of the forward model. In addition, the Adam algorithm and a chromophore- and fluence-dependent step size were used to address the large scale of the inverse problem (>10 million variables). High resolution 3-D maps of absolute chromophore concentrations and their ratios were recovered with high accuracy. The limited-view correction was found to greatly improve the agreement between true and recovered concentrations, while having a negligible effect on the accuracy of the recovered concentration ratios. Current limitations of the method lie in an insufficient compensation of image reconstruction artifacts, which can corrupt the parameter values recovered from regions containing a discrete absorber, and *a priori* knowledge of the scattering coefficient. The adverse effect of artifacts can be compensated by incorporating a model of the acoustic propagation and detection into the forward model, albeit at the expense of computation speed. While the method also enables the recovery of the scattering coefficient in principle, additional methods, such as multiple source illumination, may increase the sensitivity to this parameter.

## Conflict of interest

The authors declare that there are no conflicts of interest related to this article.
